# Piezo-surgery in periodontics

**DOI:** 10.6026/973206300181177

**Published:** 2022-12-31

**Authors:** Chatterjee Shubhangini, Rajasekar Arvina

**Affiliations:** 1 Saveetha Dental College and Hospitals, Saveetha Institute of Medical and Technical Sciences, Saveetha University, Chennai - 600077

**Keywords:** Piezoelectric device, Piezosurgery, Osseous surgery

## Abstract

Periodontitis is a chronic inflammatory disease of the tooth-supporting structures. The treatment of this condition is primarily based on the removal of local factors and the restoration of the bony architecture. Furthermore, in the modern era of dentistry, successful implant therapy frequently necessitates strong osseous support. Traditionally, osseous surgery has been performed using either manual or motor-driven instruments. Both methods, however, have advantages and disadvantages. A novel surgical approach utilising a piezoelectric device has recently been introduced in the field of periodontology and oral implantology. This narrative review article discusses the numerous applications of this novel technique of piezosurgery in periodontology.

## Background:

Periodontitis is a chronic condition of the oral cavity affecting millions of people worldwide. Periodontitis is a periodontal tissue inflammatory disease characterized by a loss of support for the affected teeth, specifically periodontal ligament fibres and the bone into which they are inserted [1]. Treatment modalities for periodontitis consist of both surgical and non-surgical methods. Surgical methods of treatment for affected bone include both resective and regenerative modalities. Apart from the conventional methods of resective surgeries using hand instruments, and use of motor for bone removal, piezosurgery is a recent advancement in periodontal therapy.[2,3] Piezosurgery is a novel and cutting-edge technique for performing precise and safe osteotomies that makes use of piezoelectric ultrasonic vibrations. It was invented by Tomaso Vercelotti to overcome the limitations of traditional instruments in oral bone surgery. [4] Its first use was in preprosthetic surgery, specifically alveolar crest expansion and sinus grafting. It significantly improves dental/implant surgery, benefiting both the surgeon and the patient by reducing surgical trauma and promoting rapid healing. When the bone is very dense, motor-driven instruments are frequently used. Motor-driven instruments convert electric or pneumatic energy into mechanical cutting action by using the sharpened edges of burs or saw blades. The cutting zone of these instruments generates a significant amount of heat, which must be reduced by water irrigation. Overheating of adjacent tissues could alter or delay the healing response. [5] Reduced rotational speed reduces both frictional heat and cutting efficiency. Motorised cutting tools also reduce tactile sensitivity; slower rotational speed necessitates increased manual pressure, which increases the cutting tool's macro-vibration and further reduces sensitivity. Manual bone removal is used to remove small amounts of bone in areas with less dense mineralization. In cortical bone, manual instruments are difficult to control, especially when precise osteotomies are required. They are primarily used for the gross cutting of large bone segments.

The word "piezo" is derived from the Greek word piezein, which means "to press tightly, squeeze." In 1880, Jacques and Pierre Curie discovered piezoelectricity when they discovered that applying pressure to various crystals, ceramics, or bones created electricity. This piezo effect is based on basic electric and mechanical interactions and phenomena such as electric field strength, polarisation, tension, and extension in the crystalline field, which states that deformation in crystals caused by passing electric current results in ultrasonic frequency oscillations. The obtained vibrations are amplified and transferred to a vibration tip, which when applied with slight pressure to bone tissue causes cavitation, which is a cutting effect that only occurs on mineralized tissue. [6] Gabriel Lippmann discovered the converse piezoelectric effect in 1881, which was later investigated by other scientists.[4].In 1953, Catuna invented an ultrasonic drill for cavity preparation on human teeth and published a paper on the effects of ultrasound on dental hard tissues. [7] Richman was the first to describe the use of an ultrasonic chisel without slurry to remove bone and resect roots in apicoectomies in 1957. [8] In 1960, Mazzarow reported the use of an ultrasonic scalpel-like blade to directly cut osseous tissue. [9] Mcfall et al. (1961) evaluated the difference in healing by comparing rotating instruments and oscillating scalpel blades and discovered slow healing with no severe complications when using these scalpel blades. [10] Horton et al. stated in 1980 that using an ultrasonic device improved bone regeneration. [11] One year later, he assessed the clinical utility of ultrasonic instrumentation in bone removal surgery and observed mineralized tissue removal with ease and efficiency, as well as patient acceptance with no complications. [12] Torella reported in 1998 that an ultrasonic generator was superior to a magnetostrictive device for cutting because it was more efficient and caused less bone destruction. [13] Dr. Thomas Vercelloti invented piezoelectric bone surgery in collaboration with Mectron Spa in 1999, and it was published in 2000. [14] Piezosurgery® was first used in 2001. The device was approved for commercial use in Germany in 2002. Vercelloti discovered the optimal frequency method for endodontic, orthopaedic, neurologic, periodontal, and oral and maxillofacial surgery in 2003. [14, 15,16] In 2005, the US Food and Drug Administration extended the use of ultrasonics in dentistry to include bone surgery. [5]

Ultrasonics is a branch of acoustics that deals with sound vibrations at frequencies above the audible level, i.e., >20 kHz, where sonic is a high-amplitude ultrasound wave produced by three different methods such as a) Mechanical Method (up to 100 kHz) b) Magneto static Method (up to 25 kHz) c) 25-50 kHz - Piezoelectric effect. The piezoelectric effect is used in piezo surgery to convert mechanical energy in the form of tension and compression into electric energy. Piezoelectric ultrasonic frequency is produced by passing an electric current from a generator through piezo-ceramic rings, causing them to deform. In dental applications, the ultrasonic frequency typically ranges between 24 and 36 kHz and is capable of cutting mineralized tissue. As a result of the ring's deformation, the accumulating movement causes a vibration in the transducer, which produces the ultrasound output. These waves are transmitted to the hand piece tip, also known as an insert, where longitudinal movement occurs, resulting in osseous tissue cutting by microscopic bone shattering. [17] The transducer is an essential component of the instrument system because it contains a piezoelectric element that converts electrical signals into mechanical vibrations and mechanical vibrations back into electric signals. Cavitation is the micro boiling phenomenon that occurs in liquids on any solid-liquid interface vibrating at an intermediate frequency, resulting in a rupture of molecular cohesion in liquids and the appearance of zones of depression that fill up with vapour until they form bubbles on the verge of imploding. Cavitation occurs in the case of detartrating tools when the water spray comes into contact with an insert vibrating at an intermediate frequency. This phenomenon maintains good visibility in the field of operation during ultrasonic osteotomy procedures by dispersing coolant as an aerosol and providing hemostasis. The cavitation effect also has an antibacterial effect by fragmenting the bacterial cell wall, which aids in bone surgery predictability and morbidity.

## Indications:

(1) Soft tissue debridement, (2) root surface smoothening, (3) bone grafting, (4) implant site preparation, (5) implant removal, (6) sinus lifting procedure, (7) retrograde root canal preparation, (8) apicectomy, (9) cystectomy, 10) extraction of ankylosed teeth, and (11) orthodontic surgeries [18]

## Contraindications:

There are no absolute contraindications. Relative Contraindications: (1) Patients with cardiomyopathy, (2) Patients with uncontrolled diabetes, (3) Patients undergoing radiotherapy, (4) Patients with metal/ceramic crowns, and (5) Patients with pacemakers

## Applications in periodontology and oral implantology:

To avoid damaging the tooth surface, supra and subgingival calculus deposits and stains on teeth, periodontal pocket lavage with simultaneous ultrasonic tip movement, scaling, root planing and crown lengthening, periodontal ostectomy and osteoplasty procedures necessitate the careful removal of small quantities of bone adjacent to exposed root surfaces.[19] The piezosurgery device is used to develop positive, physiologic bone support architecture for the involved teeth.The piezosurgery device can be used for soft-tissue debridement after an incision through retained periosteum to remove the secondary flap. The piezosurgery device can be used to debride the field of residual soft tissue and for root surface scaling to ensure thorough calculus removal by switching to a thin, tapered tip and adjusting the power setting. The piezo surgery device is used to create positive architecture for pocket elimination surgery during osteoplasty and ostectomy. [19] The device allows for precise bone removal while minimizing the risk of injury to the underlying root surfaces. Smoothing the root surfaces and bony margins with a specific ultrasonic insert, PP1, results in a clean field with ideal bony architecture ready for flap closure. The piezosurgery device is used to repair an infrabony periodontal defect with bone grafting. Autogenous bone can be easily harvested from nearby sites with minimal trauma and, as a result, minimal postoperative effects. [11] Implant site preparation, implant removal [14], bone harvesting, bone grafting, and sinus lifts can all be performed with much less soft tissue trauma.

## Advantages:

[1] It has an optimal vibration frequency for the mineralized tissue required during cutting and keeps the bone clean while preventing overheating.

[2] The use of a modulated ultrasonic frequency allows for highly precise and safe cutting of hard tissue while preserving adjacent soft tissue and nerves, making piezosurgery superior to conventional rotating instruments.

[3] The force required to make cuts is far less than that required with a drill or oscillating saw, allowing for maximum surgical control with piezosurgery.

[4] Provides direct visibility during the osteotomy procedure, reducing the risk to adjacent oral soft tissues. [20]

[5] Piezosurgery units can cut highly mineralized tissue and are three times more powerful than standard ultrasonic units.

[6] Because it does not thermally traumatise bone, there is little intraoperative bleeding, and the wound heals quickly after surgery.

[7] Using piezosurgery tips, the harvested bone can be precisely modified and shaped to fit the recipient.

[8] The cavitation effect caused by the interaction of the irrigant solution and the oscillating tips clears the surgical site during osteotomy.

## Limitations:

[1] This type of procedure requires dexterity and a gentle touch, as well as a different learning curve

[2] A rise in working pressure above a certain threshold stifles the insert's vibrations, converting the energy into heat. As a result, using a piezosurgery handpiece at a higher speed and lower pressure is the most effective method.

[3] Increased operative time when compared to traditional cutting tools.

[4] Difficulties encountered in deeper osteotomies sites due to a lack of inserts of adequate length and thickness to avoid increasing hand pressure and preventing insert micro vibration. 

[5] Inserts wear out quickly, so it is recommended that no more than ten uses be made in bone surgery because uncontrolled heat can break or damage tissues. [17] 

[6] Not economically feasible 

## Conclusion:

Piezosurgery is a sophisticated and safe bone-cutting technique that uses ultrasonic micro vibrations to produce extremely predictable results. Precision bone cutting, soft-tissue protection, minimum blood loss, a clean surgical field, minimal sound and vibration, and good patient comfort with optimal safety to the tooth structures are all advantages of piezosurgery. Despite the increased intraoperative time and the requirement for professional skill and training, piezosurgery has transformed difficult treatments into simple and practical procedures, especially in remote areas. Following piezosurgery, post-operative recovery and wound healing are favourable, allowing for optimum bone regeneration. With new technological advancements, the piezo surgical device will be a promising modality with a wide range of applications in dentistry.
